# Effects of BRCA2 deficiency on telomere recombination in non-ALT and ALT cells

**DOI:** 10.1186/2041-9414-2-9

**Published:** 2011-12-09

**Authors:** Ester Sapir, Yaghoub Gozaly-Chianea, Suliman Al-Wahiby, Sainu Ravindran, Hemad Yasaei, Predrag Slijepcevic

**Affiliations:** 1 Brunel Institute of Cancer Genetics and Pharmacogenomics, Division of Biosciences, School of Health Sciences & Social Care, Brunel University, Uxbridge, Middlesex, UB8 3PH, UK; 2 Children Hospital of Orange County, 455 South Main Street, Orange, California, CA 92868, USA

## Abstract

**Background:**

Recent studies suggest that BRCA2 affects telomere maintenance. Interestingly, anti cancer treatments that involve BRCA2 and telomerase individually are currently being explored. In the light of the above recent studies their combinatorial targeting may be justified in the development of future treatments. In order to investigate effects of BRCA2 that can be explored for this combinatorial targeting we focused on the analysis of recombination rates at telomeres by monitoring T-SCEs (Telomere Sister Chromatid Exchanges).

**Results:**

We observed a significant increase in T-SCE frequencies in four BRCA2 defective human cell lines thus suggesting that BRCA2 suppresses recombination at telomeres. To test this hypothesis further we analyzed T-SCE frequencies in a set of Chinese hamster cell lines with or without functional BRCA2. Our results indicate that introduction of functional BRCA2 normalizes frequencies of T-SCEs thus supporting the notion that BRCA2 suppresses recombination at telomeres. Given that ALT (Alternative Lengthening of Telomeres) positive cells maintain telomeres by recombination we investigated the effect of BRCA2 depletion in these cells. Our results show that this depletion causes a dramatic reduction in T-SCE frequencies in ALT positive cells, but not in non-ALT cells.

**Conclusion:**

BRCA2 suppresses recombination at telomeres in cells that maintain them by conventional mechanisms. Furthermore, BRCA2 depletion in ALT positive cells reduces high levels of T-SCEs normally found in these cells. Our results could be potentially important for refining telomerase-based anti-cancer therapies.

## Background

Telomeres are specialized nucleo-protein structures involved in chromosome end protection. This protective function requires an intricate coordination between the mechanisms that maintain telomere structure and DNA damage response mechanisms [1]. The importance of the above coordination is highlighted by the fact that defects in numerous DNA damage response proteins have acute effects on telomere maintenance mechanisms. Selected examples include proteins such as Ku and DNA-PKcs involved in DNA double strand break (DSB) repair by non-homologous end joining (NHEJ) [2], ATM, a protein responsible for DNA damage signalling [3], MRN, a complex consisting of MRE11, RAD50 and NBS proteins responsible for DSB sensing [4] and ERCC1/XPF responsible for nucleotide excision repair (NER) [5].

Two recent studies revealed another DNA damage response protein that affects telomere maintenance, namely BRCA2 [6,7]. BRCA2 associates with telomeres during S and G2 phases of the cell cycle and mediates access of RAD51, a homologous recombination protein, to telomeres [6]. A variety of telomere dysfunction phenotypes were observed when BRCA2 was defective including telomere shortening, telomere fragility, TIFs (Telomere dysfunction Induced Foci), and increased frequencies of T-SCEs (Telomere Sister Chromatid Exchanges) [6,7]. These findings are important because BRCA2 is the first protein directly involved in human cancer with a clear role in telomere maintenance. Therefore, the findings may be relevant for both understanding the role of BRCA2 in tumorigenesis and developing novel therapeutic approaches. A number of interventions targeting telomerase in cancer cells have been proposed and some of these are undergoing clinical trials [8]. Therapeutic approaches based on BRCA2 targeting are mainly focused on exploring unusual sensitivity of BRCA2 defective tumors to Poly(ADP)ribose polymerase (PARP) inhibitors [9,10]. Targeting telomerase and BRCA2 together may be a potential future avenue for refining existing therapeutic attempts (see below).

In this study we investigated effects of BRCA2 on recombination rates at telomeres by analysing T-SCE frequencies. T-SCEs represent a marker of recombination at telomeres. Our results show that BRCA2 suppresses recombination at telomeres in cells that maintain them by conventional mechanisms. By contrast, ALT (Alternative Lengthening of Telomeres) positive cells require BRCA2 as the frequencies of T-SCEs, one of the key ALT markers, are dramatically reduced following BRCA2 knock-down.

## Results and discussion

**Figure 1:**
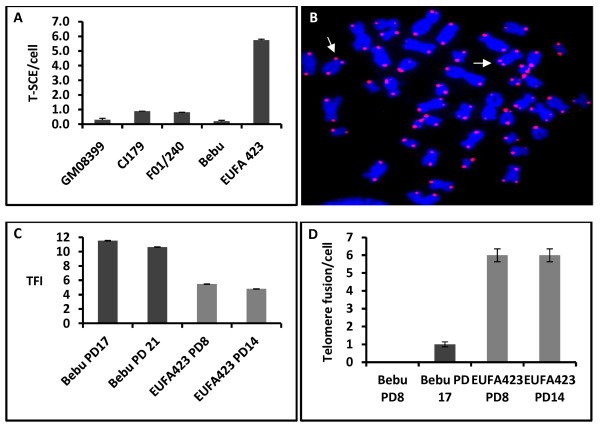
**Analysis of telomere maintenance in various cell lines**. A. Frequencies of T-SCEs (event/metaphase) in 5 cell lines. A minimum of 30 metaphases/cell line were analyzed in two independent experiments. B. Examples of T-SCEs in a metaphase cell from the EUFA423 cell line. Some of T-SCEs are indicated by arrows. C. Telomere length analysis by Q-FISH. TFI - telomere fluorescence intensity (arbitrary units). PD - population doubling. D. Frequencies of chromosome fusions. Error bars represent SEM in A and D and SD in C.

To investigate effects of BRCA2 dysfunction on recombination at telomeres we analysed T-SCE frequencies in a primary fibroblast cell line, EUFA423, obtained from a patient with biallelic mutations in BRCA2 (exons 15 and 27) leading to truncated proteins [11]. We used two primary fibroblast cell lines from normal individuals as controls (Bebu and GM08399). In addition, we used two primary fibroblast cell lines defective in Artemis (CJ179 and F01-240) [12], a NHEJ protein, to exclude the possibility that the effect of BRCA2 may be a consequence of a defective DNA damage response and thus non-specific. Our analysis revealed a statistically significant difference (p < 0.05) in T-SCE frequencies between the EUFA423 cell line and all other cell lines (Figure 1A). Normal, control cell lines and Artemis defective cell lines showed < 1 T-SCE/cell (Figure 1A). In contrast, EUFA423 cells had ~ 6 T-SCEs/cell (Figure 1A). Examples of T-SCEs observed in the above cell lines are shown in Figure 1B.

We have also analyzed telomere length and telomere function in the EUFA423 cell line and one control primary cell line, Bebu. In line with recently published studies [6,7] EUFA423 cells exhibited significantly shorter telomeres and significantly elevated frequencies of telomeric fusions relative to the control counterparts (Figure 1C &1D). Furthermore, we analyzed frequencies of TIFs in both cell lines. The EUFA423 cell line exhibited a ten-fold higher frequencies of TIFs (49% cells TIF positive; n = 136; PD 20) in comparison with the control Bebu cells (5% cells TIF positive; n = 106; PD 25). This difference was statistically significant (p < 0.05; t-test).

**Figure 2:**
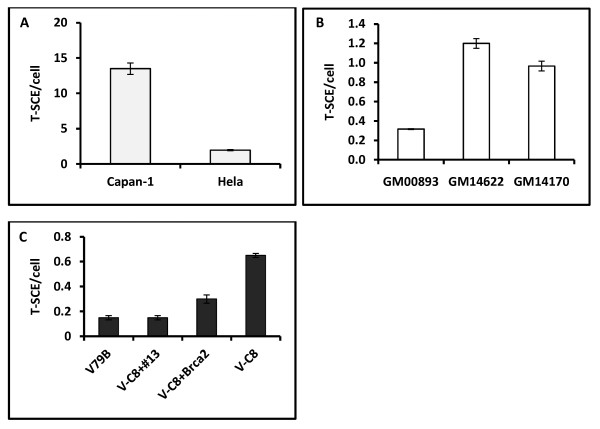
**Frequencies of T-SCEs in different sets of cell lines**. A. Frequencies of T-SCE/cell in Capan-1 and HeLa cells. A minimum of 50 metaphases/cell line have been analyzed. Error bars represent SD. B. Frequenceis of T-SCEs in three lymphoblastoid cell lines: GM00893 (normal control) GM14622 and GM14170 (cell lines established from BRCA2 carriers). A minimum of 50 metaphases/per cell line analyzed. Error bars represent SEM. C. Frequencies of T-SCEs in a set of Chinese hamster cell lines: V79B (normal control); V-C8 (BRCA2 defective, see the text for details), V-C8+#13 (the V-C8 cell line in which the BRCA2 defect was complemented by introducing human chromosome 13 containing the BRCA2 gene) and V-C8+Brca2 (the V-C8 cell line transfetced with a BAC containing a copy of murine Brca2). A minimum of 50 cells/per cell line analyzed. Error bars represent SEM.

To test whether the elevated T-SCE frequency observed in EUFA423 cells is specific to the BRCA2 defect we used additional BRCA2 defective cell lines. These included: (i) Capan 1, the pancreatic carcinoma cell line completely lacking one BRCA2 allele and containing a truncation (6174delT) mutation in the other allele leading to a truncated protein product [13] and (ii) two lymphoblastoid cell lines, GM14622 and GM14170, from patients with constitutive *BRCA2 *mutations (frameshift mutation 6503delTT in exon 11, leading to a truncation at codon 2099 and a 1bp deletion at nucleotide 6174 in exon 11 resulting in a frameshift beginning at codon 1982 and terminating at codon 2003) http://ccr.coriell.org/Sections/BrowseCatalog/DiseaseDetail.aspx?PgId=403&omim=BRC60018&coll=. The CO-FISH analysis revealed a sevenl-fold higher T-SCE frequency in the Capan 1 cell line than in the control HeLa cell line (Figure 2A). Similarly, T-SCE frequencies were several times higher in the two BRCA2 defective lymphoblastiod cell lines in comparison with the control cell line (Figure 2B). Therefore, elevated recombination rates at telomeres observed in four different BRCA2 defective cell lines suggest that BRCA2 suppresses recombination at telomeres. This is in line with a recent study reporting elevated frequencies of T-SCEs in BRCA2 heterozygous cell lines [7].

To further test the hypothesis that BRCA2 suppresses recombination at telomeres we used a set of Chinese hamster cell lines including a BRCA2 defective cell line, V-C8, which shows two nonsense mutations in exons 15 and 16 of the Chinese hamster *Brca2 *gene resulting in two truncated proteins [14], and two isogenic cell lines in which the BRCA2 defect was corrected either as a result of introducing a BAC with a functional copy of the murine *Brca2 *gene (V-C8+Brca2), or human chromosome 13 containing functional *BRCA2 *(V-C8+#13) [15]. The parental V79B cell line with functional *Brca2 *was used as a control cell line. As expected, V-C8 cells, showed several-fold higher frequencies of T-SCEs relative to control V79 cells (Figure 2C). However, the correction of the BRCA2 defect resulted in significant reduction of T-SCE frequencies (p < 0.05) (Figure 2C). In the case of the V-C8+#13 cell line the frequency of T-SCEs was the same as that observed in the V79 cell line (Figure 2C). The V-C8+Brca2 cell line showed somewhat higher frequency of T-SCE relative to V79 cell line but still significantly lower than in the V-C8 cell line (Figure 2C). These results suggest that correction of the BRCA2 defect leads to normalization of T-SCE frequencies, thus further supporting the hypothesis that BRCA2 suppresses recombination at telomeres.

**Figure 3:**
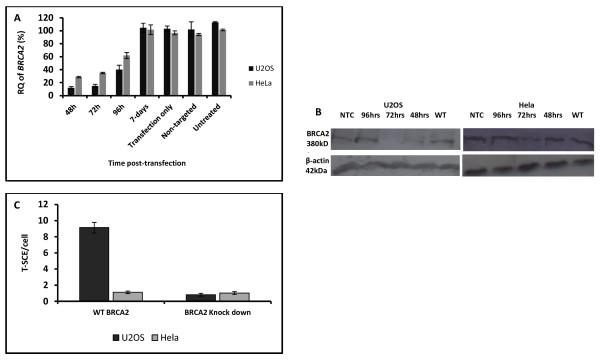
**BRCA2 knock-down using siRNA oligonucleotides in U2OS and HeLa cells**. A. Relative quantities (RQ) of BRCA2 mRNA assessed by quantitative real time PCR after various times following transfection with siRNA oligonucleotides specific for BRCA2. Error bars represent SD. B. Western blot analysis of BRCA2 expression following transfection with siRNA oligonucleotides. C. Frequencies of T-SCEs in U2OS and HeLa cells after BRCA2 depletion. T-SCE frequencies were measured 72 hrs after transfection with siRNA oligonucleotides. A minimum of 50 cells have been analyzed in two independent experiments. Error bars represent SEM.

The above findings are interesting from the perspective of the ALT mechanisms for telomere maintenance. ALT relies on recombination [16] and previous studies have shown that BRCA2 associates with APBs (ALT-associated Promyelocytic leukemia Bodies) [17] and that the knock-down of FANCA and FANCD2 proteins, both involved in the same pathway as FANCD1/BRCA2, causes reduction of T-SCE frequencies in ALT positive cells [18]. Therefore, we investigated the effect of BRCA2 depletion in ALT positive cells. To this end we selected an ALT positive cell line, U2OS, and determined T-SCE frequency, one of the ALT markers [16], relative to the control non-ALT cell line, HeLa. As expected U2OS cells showed approximately 10 fold higher frequencies of T-SCEs relative to HeLa cells (results not shown). We then knocked-down BRCA2 expression in these two cell lines using siRNA oligonucleotides specific for BRCA2. To verify the knock-down we used quantitative RT-PCR and Western blot. The RT-PCR results show that this procedure resulted in 70%-85% BRCA2 knockdown 48h and 72h after transfection (Figure 3A). A similar pattern was observed by Western blot (Figure 3B).

Having verified the BRCA2 knockdown in the above cell lines we investigated its effect on T-SCE frequencies. In a pilot experiment we found that that the BRC2 knock-down slowed the cell cycle progression in both cell lines and caused a low mitotic index. This precluded adequate yield of metaphase cells for CO-FISH analysis 48 h after transfection (results not shown). As a result, we performed the CO-FISH procedure on U2OS and HeLa cells 72 h following BRCA2 knock-down. The BRCA2 knock-down caused a dramatic, 9-fold reduction (p-value = 7.5 × 10^-12^), in T-SCE frequencies in ALT positive, U2OS cells, leading to the levels of T-SCEs observed in non-ALT HeLa cells (Figure 3C). Similar results were reported when FANCA and FANCD2 were depleted in ALT positive cells [18]. The differences in T-SCE frequencies in non-ALT HeLa cells with and without functional BRCA2 were not statistically significantly different (p-value = 0.69). This suggests that the transient BRCA2 knockdown is not sufficient to cause elevated rates of T-SCEs in HeLa cells.

**Figure 4:**
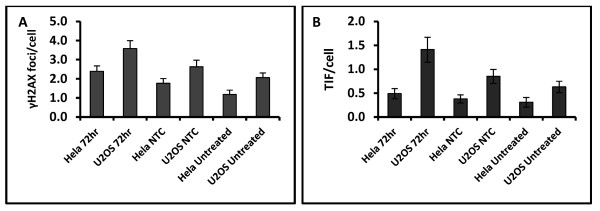
**DNA damage response and telomere analysis after BRCA2 depletion**. A. Frequencies of γH2AX foci measured 72 hrs post transfection in U2OS and Hela. A total of 50 cells were analysed per point in two independent experiments. Error bars represent SEM B. Frequencies of TIFs in U2OS and Hela cell lines analysed 72 hrs post transfection. A total of 50 cells were analysed per point in two independent experiments. Error bars represent SEM. NTC (non-template control) represent scrambled siRNA.

Interestingly, we have also observed some chromosome fusion events after the BRCA2 knock-down in ALT positive cells (results not shown). The presence of chromosome fusion events is a sign of telomere dysfunction. In order to investigate this further we analyzed frequencies of TIFs in U2OS and HeLa cells after BRCA2 knock-down (72 h after transfection). Since the TIF protocol simultaneously detects DNA damage foci and telomeres (see Material and methods) we calculated frequencies of DNA damage foci as detected by an antibody against γH2AX (Figure 4A). The BRCA2 depletion resulted in increased frequencies of DNA damage in both HeLa and U2OS cell lines (Figure 4A). As expected, U2OS cells showed significant increase in TIF frequencies after BRCA2 knock-down suggesting a two-fold effect of BRCA2 on telomere maintenance in the ALT positive cells; reduction in T-SCE frequencies and elevated frequencies of dysfunctional telomeres. Increase in dysfunctional telomeres after BRCA2 knockdown was also observed in HeLa cells (Figure 4B) thus confirming results reported in recent studies [6,7].

Taken together our results reveal two novel features of BRCA2. First, BRCA2 suppresses recombination at telomeres in cells which maintain telomeres by conventional mechanisms. Second, in cells which maintain telomeres by ALT mechanisms BRCA2 has the opposite effect as the frequency of an ALT marker, T-SCEs, is dramatically reduced following the BRCA2 depletion. Given that one of the drawbacks associated with telomerase-based cancer therapy is the activation of ALT [8] identification of proteins that are essential for this mechanism is important. It has been reported previously that the depletion of the MRN complex [19], FANCD2 and FANCA [18] disrupts the ALT pathway. Therefore, the use of BRCA2 depletion, or depletion of proteins such as MRN, FANCD2 or FANCA, in combination with anti telomerase therapeutic approaches may prevent activation of the ALT pathway and thus make anti-telomerase mediated tumor cell killing more effective. Furthermore, BRCA2 defective tumors may respond well to telomerase inhibition as these tumors may not be able to activate ALT.

## Methods

### Tissue culture

The normal human primary fibroblast cell lines, GM08399 (purchased from Coriell Cell Repositories), Bebu (obtained from Dr Hans Joenje, Amsterdam), Artemis defective primary fibroblast cell lines FO1/240 and CJ170 (obtained from Dr Penny Jeggo, University of Sussex) and BRCA2 defective primary fibroblast cell line EUFA423 (obtained from Dr Hans Joenje) were grown in D-MEM medium supplemented with 10% foetal calf serum (FCS) and antibiotics in the atmosphere of 10% CO_2_. HeLa and Capan 1 cell lines were grown under the same conditions.

Lymphoblastoid cell lines GM14622 and GM14170 established from BRCA2 heterozygote patients and a normal lymphoblastoid cell line GM00893 were purchased from Coriell Cell Repositories and grown in RPMI-1640 Medium supplemented with 10% FCS and antibiotics. All Chinese hamster cell lines (a gift from Dr M. Zdzienicka, Leiden University) were grown in F10 medium supplemented with 10% FCS and antibiotics.

### Chromosome orientation fluorescence in situ hybridization (CO-FISH), quantitative(Q)-FISH and TIF

CO-FISH was performed as described [20]. Briefly, cells were incubated for 24 h in the presence of Brdu and Brdc (3:1) (Sigma Aldrich) at a concentration of 1 × 10^-5^. Microscope slides containing chromosome preparation were stained with Hoechst 33258 (0.5 μg/ml; Sigma Aldrich) for 15 min at room temperature, and exposed to 365 nm UV light source (Stratelinker 1800) for 30 min. Enzymatic digestion of the Brdu/Brdc substituted DNA strand was performed with 3 U/ml of Exonuclease III (Promega) in the buffer supplied by the manufacturer. In situ hybridization was performed using the PNA oligonucleotide CCCTAA labelled with FITC (PE Biosystem) as described (10). Images of metaphase cells were captured using a Zeiss Axioplan microscope equipped with the JAI CCD camera and Imaging Associates software. Q-FISH and TIF protocols were performed as described previously [12,21]. Where the frequency of γH2AX is presented it was extracted from the TIF results as the TIF protocol simultaneously detects γH2AX foci and telomeres [21].

### siRNA transfection and real time quantitative reverse-transcription (qRT)-PCR

Cells were plated at a seeding density of 0.045 × 10^6 ^cells/500 μl roughly equating to 40% confluency in a 24-well plate. All siRNA transfections were done in duplicate using ON-TARGET plus SMARTpool (Thermo Fisher Scientific, Dharmacon Products) targeting *BRCA2 *gene (NM-000059) and a non-targeting (scrambled) siRNA as a negative control at a final concentrationof 50 nM. Transfection was performed using 8 μl of jetPRIME™transfection reagent (Polyplus transfection). Total RNA was extracted at various time points as shown in the results section. The sequences of the human *BRCA2 *gene siRNA in the SMARTpool were as recommended by the manufacturer.

A two step qRT- PCR was performed using SYBR green 1 dye (Applied Biosystems) and expression of mRNA quantified in real-time with an ABI prism 7900HT sequence detection system (Applied Biosystems). The relative gene expression of *BRCA2 *was measured against the endogenous *GAPDH *gene and calculated using ΔCt. The sequence of *BRCA2 *and *GAPDH *primers were: forward 5'-AATGCCCCATCGATTGGTC-3' and reverse 5'-AGCCCCTAAACCCCACTTCAT-3', forward 5'-GAAGGTGAAGGTCGGAGT-3' and reverse 5'-GAAGATGG TGATGGGATTTC-3' respectively.

### Western blot

Western blotting has been carried out as described previously (12). Briefly, cells were lysed with 900 μl of lysis buffer (5 × sample buffer; 10% (v/v) sodium dodecyl sulphate, 250 mM tris pH 8.0, 50% (v/v) glycerol, 0.01% (w/v) bromophenol blue). In addition, 50 μl of protease inhibitor (Roche) plus 50 μl of beta-mercaptoethanol was added and left for at least one minute followed by mechanical shearing using a 1 ml syringe and a 23 g needle. Samples were spun at 13,000 RPM for 5 minutes at 4°C. Proteins were quantified usingCB-X protein assay (G-Biosciences). Equal concentrations of 50 μg/50 μl were loaded onto a 4% until the high molecular weight protein marker (Invitrogen) was well separated. Following a wet blotting transfer to a polyvinylidine fluoride (PVDF) membrane and blocking with 5% (w/v) semi-skimmed milk (Marvel), the membrane was incubated with the mouse monoclonal anti-BRCA2 antibody (Calbiochem) and β-actin (Abcam, Cambridge, UK) in a 1:500 and 1:1000 dilution respectively at 4°C overnight followed by incubation with secondary anti-mouse IgG-HRP conjugated (Sigma-Aldrich) antibody at a concentration of 1:5000.

## Competing interests

The authors declare that they have no competing interests.

## Authors' contributions

ES carried out BRCA2 knock-down, verified it by RT-PCR and monitored frequencies of T-SCEs. YGC analyzed Chinese hamster cell lines and carried out Western blot experiments. SAW carried out Q-FISH and telomere fusions analysis. SR participated in T-SCE analysis. HY participated in RT-PCR analysis. PS designed experiments and wrote the manuscript. All authors read and approved the final manuscript.
